# Ligation of CM1 enhances apoptosis of lung cancer cells through different mechanisms in conformity with EGFR mutation

**DOI:** 10.3892/ijo.2012.1731

**Published:** 2012-12-07

**Authors:** HYUN-KYUNG LEE, GA BIN PARK, YEONG SEOK KIM, HYUNKEUN SONG, V. COURTNEY BROADDUS, DAE YOUNG HUR

**Affiliations:** 1 Department of Internal Medicine, Inje University Busan Paik Hospital; Busan 614-735, Republic of Korea;; 2 Department of Anatomy and Research Center for Tumor Immunology, Inje University College of Medicine, Busan 614-735, Republic of Korea;; 3 Lung Biology Center, San Francisco General Hospital, University of California San Francisco, San Francisco, CA 94110, USA

**Keywords:** CM1, lung cancer, EGFR mutation, apoptosis

## Abstract

Although remarkable developments in lung cancer treatments have been made, lung cancer remains the leading cause of cancer mortality worldwide. Epidermal growth factor receptor (EGFR) is occasionally mutated in non-small cell lung cancer and heterogeneity in treatment response results from different EGFR mutations. In the present study, we found that centrocyte/centroblast marker 1 (CM1), previously reported as a possible apoptosis inducer of B lymphoma cells, is expressed on both A549 with wild-type EGFR and HCC827 with mutant EGFR lung cancer cells. Ligation of CM1 with anti-CM1 mAb enhanced apoptosis in both lung cancer cell lines through generation of reactive oxygen species (ROS) and disruption of mitochondrial membrane potential, however, the signaling mechanisms differed from each other. Further studies to investigate the signaling mechanisms identified that ligation of CM1-induced apoptosis in A549 cell involved FasL expression, caspase-8, ERK1/2 and Akt kinase, whereas apoptosis of HCC827 cells was induced through caspase-9, JNK and c-jun-dependent pathways. Taken together, we suggest that CM1 could be developed as a therapeutic target of lung cancer regardless of EGFR mutation status.

## Introduction

Lung cancer is the leading cause of cancer mortality worldwide. Although remarkable developments in various cancer treatments have been made, the overall 5-year relative survival rate of patients with lung cancer remains less than 20% in most countries (1–3). Moreover, in the advanced stages of lung cancer, only palliative therapies (chemotherapy and/or radiotherapy) are given as the standard of care (4). Therefore, new therapeutic agents to improve the prognosis of lung cancer are urgently needed. Various novel therapeutic strategies are currently under investigation because the clinical use of cytotoxic drugs is limited due to intrinsic or acquired resistance and toxicity (5). In addition, there has been a paradigm shift in cancer therapeutics from the use of conventional cytotoxic drugs to the use of variable molecular-targeted therapeutics including gefitinib in lung cancer or trastuzumab in breast cancer (6,7). Some candidate molecules are expressed in specific cancer cells and methods of targeting these alterations have been developed. For example, several monoclonal antibodies or small molecules that can inhibit the growth and proliferation of specific cancers are now available (8,9). A better understanding of the molecular mechanisms of targeted drug action has shed light on the treatment of lung cancer, and novel agents that target specific intracellular pathways related to the distinctive properties of cancer cells continue to be developed.

Epidermal growth factor receptor (EGFR) is occasionally mutated in non-small cell lung cancer and heterogeneity in treatment response could result from differences in EGFR mutation status (10,11). The A549 tumor-cell line with wild-type EGFR, derived from a human alveolar epithelial cell carcinoma, has been studied *in vitro* to evaluate lung cancer behavior (12). HCC827 cells are lung adenocarcinoma cells with an activating mutation in the EGFR tyrosine kinase domain (13). In the present study, we evaluated a new cell surface molecule expressed on both A549 and HCC827 cells to consider the different response dependent on EGFR mutation status.

Centrocyte/centroblast marker 1 (CM1) is a new putative germinal center marker defined by a monoclonal antibody developed against concanavalin-A-activated peripheral blood mononuclear cells (PBMCs). It was originally reported that several cancer cell lines, such as Raji, Ramos and IM-9, which originate from human B cells, express CM1 molecules on their cell membranes (14). Moreover, the expression of CM1 is induced during transformation of B cells by Epstein-Barr virus infection. Most importantly, ligation of CM1-induced apoptosis of CM1^+^ cells (15,16). These studies suggest that CM1 may be expressed on other cancer cells including lung cancer and serve as a potential target in CM1^+^ cancer cells. In this study, we investigated the expression and role of CM1 molecules in both A549 and HCC827 lung cancer cells.

## Materials and methods

### Cell preparation and culture

A549 and HCC827 cells were obtained from the American Type Culture Collection (ATCC, Rockville, MD, USA). These cells were grown and maintained in RPMI-1640 medium (HyClone, Logan, UT, USA) containing 2 mM L-glutamine, 10 U/ml penicillin, 100 *μ*g/ml streptomycin and 10% heat-inactivated fetal bovine serum (HyClone) at 37°C in a 5% CO_2_ incubator.

### Flow cytometry

The cells were washed twice with phosphate-buffered saline (PBS) and incubated with either fluorescein isothiocyanate (FITC)-conjugated mouse anti-human CM1 antibody (a gift from Dr W.J. Lee, Seoul National University, Seoul, Korea), mouse anti-human Fas antibody (BD Pharmingen, San Jose, CA, USA) or mouse anti-human FasL antibody (BD Pharmingen) for 30 min on ice. The cells incubated with anti-Fas and anti-FasL antibodies were further stained with FITC-conjugated goat anti-mouse IgG antibody (Sigma, St. Louis, MO, USA) for 30 min on ice and washed twice with PBS. MOPC (IgG1) was purchased from Sigma-Aldrich (St. Louis, MO, USA) as the isotype control antibody. A FACSCalibur (BD Pharmingen) flow cytometry was used for analysis.

### Confocal microscopy

To detect surface molecules, cells were incubated with FITC-conjugated anti-CM1 antibody (mouse IgG_1_) and, to detect intracellular molecules, cells were first treated with permeabilization buffer (0.1% saponin in PBS) before incubation with various antibodies. Cells were then incubated with primary antibody against cytochrome *c* (mouse IgG_2b_, Santa Cruz Biotechnology, Santa Cruz, CA, USA), AIF (mouse IgG_2b_, Santa Cruz Biotechnology) or endoG (mouse IgG2b, Santa Cruz Biotechnology). Cells were then washed thrice with PBS, and incubated with FITC-conjugated goat anti-mouse IgG antibody (Sigma-Aldrich) for 30 min. The nucleus was stained with propidium iodide (PI, BD Pharmingen) at RT for 10 min. After being washed thrice with PBS, cells were mounted onto microscopic slides under coverslips using fluorescent mounting medium (DakoCytomation, Glostrup, Denmark). Fluorescent cells were examined by Confocal Laser-Scanning microscopy (510 META, Carl Zeiss, Jena, Germany) at ×400 magnification, and images were acquired with Confocal Microscopy Software Release 3.0 (510 META, Carl Zeiss).

### Induction of CM1-mediated signaling

For immobilization, anti-CM1 or MOPC21 (IgG1κ, isotype control antibody, Sigma-Aldrich) antibodies (50 *μ*g/ml in PBS) were incubated overnight at 4°C on a 96-well culture plate (0.1 ml/well; washed with PBS before use). Each antibody was used at various concentrations (0.625, 1.25, 2.5, 5 and 10 *μ*g/ml). A549 and HCC827 cells (5×10^5^ cells/well) were incubated in plates coated with antibodies at 37°C for 2, 4, 8 and 12 h. In some cases, cells were pretreated with z-VAD-fmk as a broad caspase inhibitor (20 *μ*M, Calbiochem, La Jolla, CA, USA), z-DEVD-fmk (N-benzyloxycarbonyl-Asp-Glu-Val-Asp-fluoromethylketone, 20 *μ*M in DMSO, a caspase-3 inhibitor), and z-IETD-fmk (N-benzyloxycarbonyl-Ile-Glu-Thr-Asp-fluoromethylketone, 20 *μ*M in DMSO, a caspase-8 inhibitor) from Calbiochem for 2 h before stimulation with anti-CM1 antibody. In some cases, NAC, a ROS inhibitor (10 mM, Sigma-Aldrich) or ZB4, anti-Fas antibody (0.5 *μ*g/ml, Abcam, Cambridge, UK), was added 1 h before stimulation with anti-CM1 antibodies. Cells were washed of all chemicals and antibodies before CM1 stimulation.

### Proliferation measurement by AlamarBlue assay

The evaluation of cell growth was determined using an AlamarBlue assay. A549 and HCC827 cells (5×10^4^ cells/well) were cultured in medium containing 10% FBS in 96-well flat bottom plates and treated with 10 *μ*g/ml anti-CM1 antibodies or MOPC (isotype control antibodies) for 48 h before adding AlamarBlue solution (Serotec Ltd, Kidlington, UK). AlamarBlue was added (10% by volume) to each well and the relative fluorescence was determined 7 h later by Fluorometer (Synergy HT; Bio-Tek Instruments Inc., Winnoski, VT, USA; excitation, 570 nm; emission, 600 nm). Experiments were performed in triplicate.

### Detection of CM1-mediated apoptosis

To evaluate the apoptosis-inducing effect of CM1, cells were analyzed for Annexin V expression by flow cytometry. Following treatment, cell were collected and washed twice with PBS and resuspended in 100 *μ*l of Annexin V binding buffer (10 mM of HEPES/NaOH pH 7.4, 140 mM of NaCl, 2.5 mM of CaCl_2_). After 2 *μ*l of FITC or PE-conjugated Annexin V (BD Pharmingen) was added, cells were incubated in the dark at RT for 15 min with gentle vortexing. Finally, 400 *μ*l of Annexin V binding buffer was added to each tube and cells were analyzed using FACSCalibur (BD Pharmingen).

### Measurement of mitochondrial membrane potential and ROS generation

Cells were pretreated with 10 *μ*M of 2′, 7′-dichlorodihydrofluorescein diacetate (DCFH-DA, Molecular Probes, Eugene, OR) for 30 min and ROS levels were assessed by the conversion of DCFH to the highly fluorescent dichlorofluorescein (DCF) in the presence of intracellular ROS. Cells were washed twice with cold PBS and then incubated with immobilized mouse anti-human CM1 antibody or the isotype control antibody. To measure mitochondrial membrane potentials, cells were collected and incubated in 100 *μ*l of PBS containing 20 nM of 3,3′-dihexyloacarbocyanine iodide (DiOC_6_, Molecular Probes) at 37°C for 15 min. Cells were then collected and washed with cold PBS twice, and ROS levels and mitochondrial membrane potential were detected by FACSCalibur (BD Pharmingen).

### Reverse transcription polymerase chain reaction

Cells were washed three times with PBS. RNA was extracted using RNeasy mini kit (Qiagen, Hilden, Germany) and cDNA was produced using RT premix (Bioneer, Daejeon, Korea). FasL cDNA was then amplified using 5′-GGT CCA TGC CTC TGG AAT GG-3′ as a forward primer and 5′-CAC ATC TGC CCA GTA GTG CA-3′ as a reverse primer. PCR amplification was also performed using specific primer sets (Bioneer) for Bcl-2 (upstream primer, 5′-GGA TTG TGG CCT TCT TTG AG; downstream primer, 5′-CAG CCA GGA GAA ATC AAA CAG, 209-bp product), Bax (upstream primer, 5′-CCA AGA AGC TGA GCG AGT GT; downstream primer, 5′-CAG CCC ATG ATG GTT CTG AT, 250-bp product) and Bad (upstream primer, 5′-CGA GTG AGC AGG AAG ACT CC; downstream primer, 5′-CTG TGC TGC CCA GAG CTT, 299-bp product). For control, a specific primer set for β-actin (upstream primer, 5′-ATC CAC GAA ACT ACC TTC AA; downstream primer, 5′-ATC CAC ACG GAG TAC TTG C) was used, which yielded a 200-bp product. PCR (25 cycle; 20 sec at 94°C, 10 sec at 60°C, 30 sec at 72°C) was performed using AccuPower PCR premix (Bioneer). PCR products were analyzed by agarose gel electrophoresis and visualized with ethidium bromide under UV light using Multiple Gel-DOC system (Fujifilm, Tokyo, Japan).

### Western blot analysis

Cells were lysed in 50 mM Tris-Cl (pH 7.5) containing 150 mM NaCl, 1% NP-40, 0.5% deoxycholic acid, 0.1% SDS and a protease inhibitor (Sigma-Aldrich) on ice for 10 min. Lysates were clarified by centrifugation at 14,000 × g for 15 min at 4°C. Protein concentrations were determined by the Bradford method. Sample-loading buffer was added, the mixture was boiled for 5 min and the proteins were then separated by electrophoresis on 10% polyacrylamide-SDS gels before being transferred to the nitrocellulose membrane by electroblotting. Blots were blocked using overnight incubation with 5% skim milk in TBS, and then incubated for 1 h at RT with primary antibodies, followed by horseradish peroxidaseconjugated secondary antibodies (Amersham Biosciences). The following primary antibodies were used: anti-caspase-8, anti-caspase-3, Bid, phospho-ERK1/2 (Thr^202^/Tyr^204^), ERK1/2, phospho-Akt (Ser^473^), Akt, caspase-9, Bcl-X_L_, phospho-JNK (Thr^183^/Tyr^185^), JNK and β-actin antibodies from Cell Signaling Technology (Beverly, MA); phospho-c-jun and c-jun from Santa Cruz Biotechnology; PARP [poly(ADP-ribose) polymerase] from Upstate Biotechnology (Lake Placid, NY). Data were analyzed using ImageJ 1.38 software.

## Results

### CM1 expression on the surface of lung cancer cells

**Figure 1.**
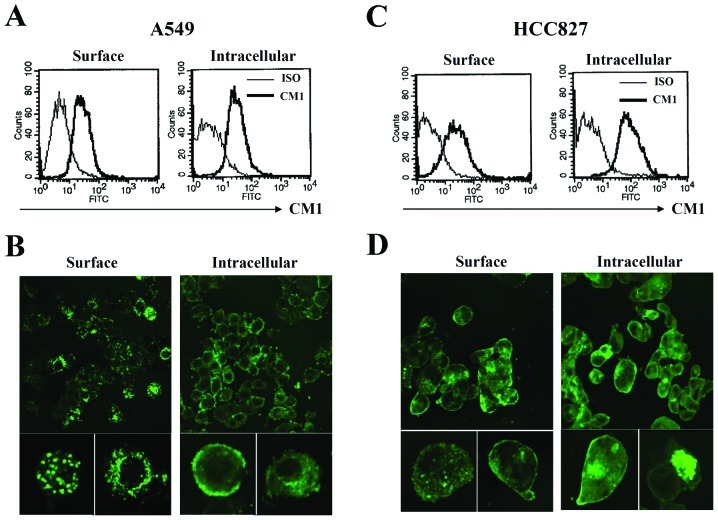
Flow cytometric and confocal microscopic analyses for the CM1 expression on (A and B) A549 and (C and D) HCC827 cells. The cells were stained with FITC-conjugated mouse anti-human CM1 antibody, and fluorescence was detected and calculated by (A and C) flow cytometry and (B and D) confocal microscope as described in Materials and methods. (A and C) The thin-line histograms represent the isotype control for anti-CM1 antibody and thick-line histograms represent cells stained with anti-CM1 antibody. (B and D) Green fluorescence portions were identified as CM1 molecules. Data are representative of three independent experiments. ISO, sample stained with FITC-conjugated isotype control antibody.

CM1 expression was evaluated on A549 and HCC827 lung cancer cells using flow cytometry and confocal microscopy. Flow cytometric analysis results showed that both A549 and HCC827 cells expressed CM1 molecules on their cell surface and intracellularly. Moreover, the CM1 expression pattern was also confirmed by confocal microscopy. Interestingly, the expression pattern of CM1 differed in the two lines: CM1 expression was clustered on the cell surface in A549 cells, but more dispersed on the cell surface in HCC827 cells (Fig. 1).

### Cross-linking of CM1 inhibits the growth of lung cancer cells

**Figure 2.**
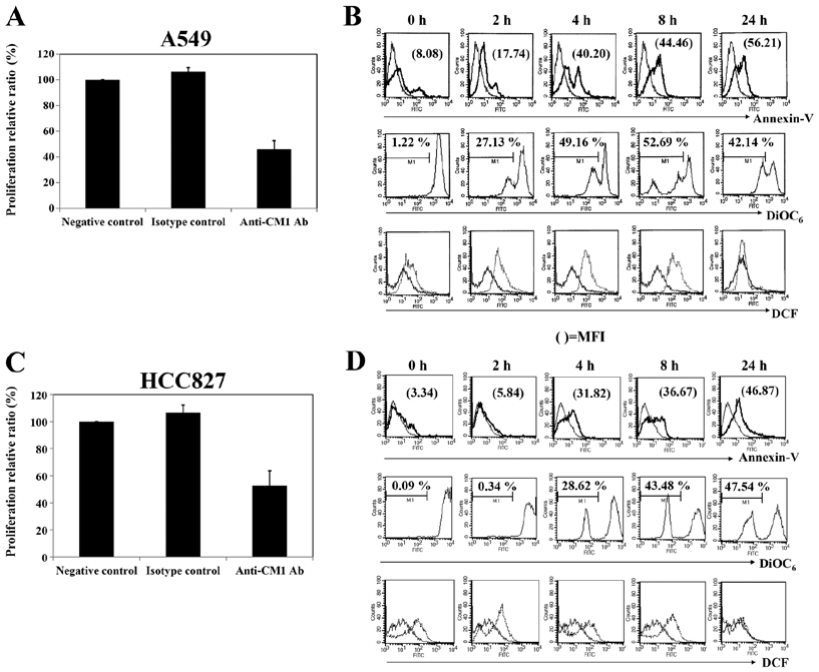
Proliferation and apoptosis in A549 and HCC827 lung cancer cells after ligation of CM1. (A and C) To measure the extent of proliferation, cells were incubated (5×10^4^ cells/well, 200 *μ*l) in an anti-CM1 or MOPC antibody-coated (2.5 *μ*g/well) plate for 48 h, and then determined by an alamarBlue assay. (B and D) To evaluate apoptosis, ROS generation and mitochondrial membrane potential disruption, cells were incubated (5×10^5^ cells/well, 200 *μ*l) in an anti-CM1 or MOPC antibody-coated (2.5 *μ*g/well) pate for 2, 4, 8 and 24 h. Cells were collected at the indicated times and washed with PBS. Twenty-four hours later, cells were stained with FITC-conjugated Annexin V as described in Materials and methods and analyzed by flow cytometry. The indicated number represents the apoptotic cell proportion (B and D, first row). The number of DiOC_6_ represents the percentage of mitochondrial membrane potential disruption of cells. The thin-line histograms represent the apoptosis of MOPC (isotype control for CM1 antibody) treated cells, and thick-line histograms represent that of anti-CM1 antibody-treated cells (B and D, second row). To measure ROS generation, cells were pre-incubated with DCFH-DA before being treated with anti-CM1 or MOPC antibody. Thin-line and dotted histograms represent ROS level of isotype controlled and anti-CM1 antibody-treated cells, respectively. The number in DCF histograms indicates the MFI (B and D, third row). Results are representative of four independent experiments.

We first determined the anti-proliferative effects of anti-CM1 mAb on both lung cancer cell lines using AlamarBlue assay after treatment with anti-CM1 mAb at various concentrations. Growth inhibition of both lung cancer cell lines was detectable following 48 h treatment using anti-CM1 mAb concentrations starting at 1 *μ*g/ml (data not shown). As shown in Fig. 2A and C, approximately 40–60% growth inhibition was achieved at a 10 *μ*g/ml concentration in both A549 and HCC827 lung cancer cells.

### CM1 stimulation induces apoptosis in both A549 and HCC827 cells

To determine whether CM1 stimulation could induce apoptosis of CM1 expressing A549 and HCC827 cells, as described in previous studies (7,8), A549 and HCC827 cells were stimulated with anti-CM1 mAb for 2, 4, 8 and 24 h. Cells were stained with FITC-labeled Annexin V and analyzed by flow cytometry. The ligation of surface CM1 molecules with anti-CM1 antibody increased the number of Annexin V positive apoptotic cells in both A549 and HCC827 cell lines. The increase of apoptotic cells in A549 was detected earlier than in HCC827 cells (Fig. 2B and D, first row).

We next characterized the molecular mechanisms underlying CM1-mediated apoptosis in these lung cancer cells. The ligation of surface CM1 molecules with anti-CM1 antibody generated ROS and disrupted mitochondrial membrane potential in both A549 and HCC827 lung cancer cells. Induction of mitochondrial membrane potential disruption in A549 cells was earlier than that in HCC827 cells (Fig. 2B and D, second and third rows). The ROS level was restored to normal in about 24 h in both cell types.

### CM1 ligation induces Fas ligand expression only on A549 cells

**Figure 3.**
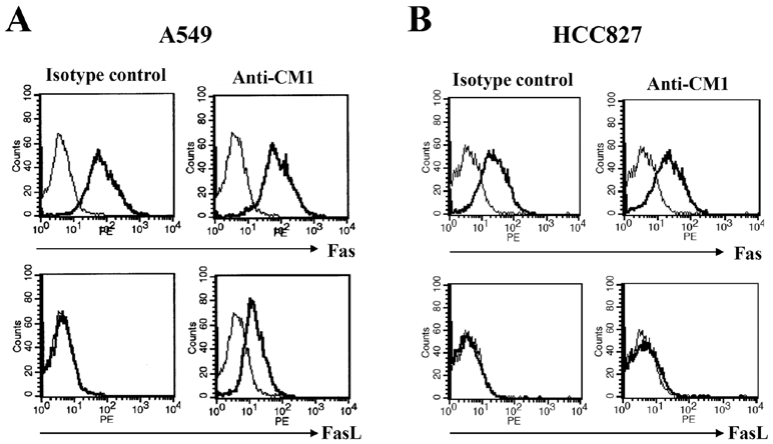
Fas and FasL expression on (A) A549 and (B) HCC827 cells after ligation of CM1. A549 cells (5×10^5^ cells/well, 200 *μ*l) were incubated in an anti-CM1 or MOPC antibody-coated (2.5 *μ*g/well) plate for 2 h. Histograms show the expression level of Fas or FasL (bold line) and isotype-matched control mAb (MOPC, solid line). Results are representative of the three independent experiments.

To examine surface molecules associated with apoptosis after CM1 ligation, flow cytometric analysis was performed for surface Fas (CD95) and Fas ligand (FasL, CD178). A549 and HCC827 lung cancer cells were incubated with immobilized anti-CM1 or MOPC antibodies (5 *μ*g/ml) for 2 h. Cells were washed and stained using FITC conjugated anti-Fas and FasL antibodies. Fas was expressed constitutively on both A549 and HCC827 cells and was unaffected by CM1 ligation. However, FasL was not expressed on unstimulated lung cancer cells, but was induced in A549 but not HCC827 cells after CM1 ligation (Fig. 3). To confirm induction of FasL on A549 cells after CM1 ligation, cells were incubated with immobilized anti-CM1 or MOPC antibody and analyzed by reverse transcription polymerase chain reaction (RT-PCR) for FasL message. FasL mRNA was increased after CM1 ligation on A549 cells (Fig. 5A, first row). We also performed these experiments in HCC827 cells, but FasL mRNA was not increased in the same conditions (data not shown).

### CM1 ligation induces Fas-mediated apoptosis on A549 cells

**Figure 4.**
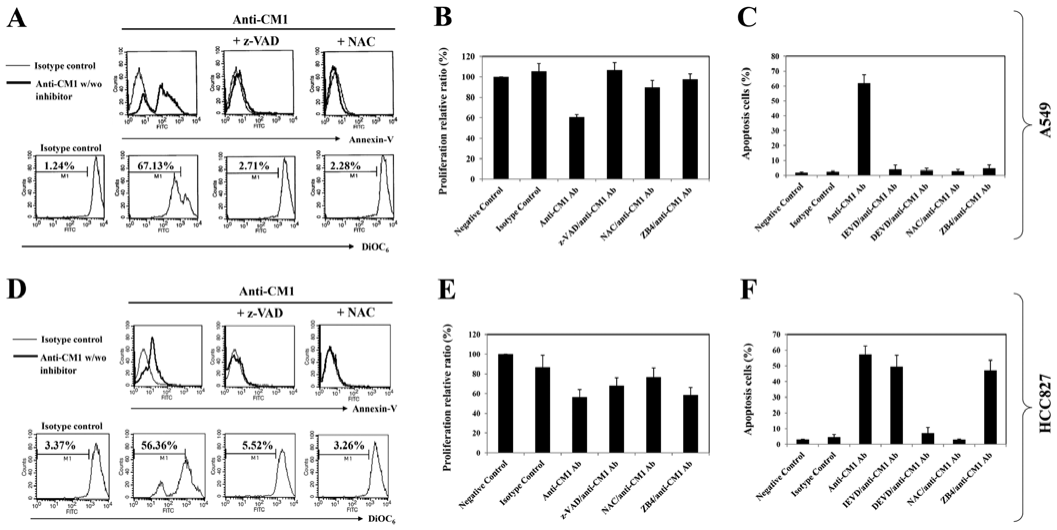
The effects of inhibition on CM1-mediated apoptosis of A549 and HCC827 lung cancer cells. Cells were pre-incubated with z-VAD-fmk (20 *μ*M, 2 h), NAC (10 mM, 1 h), z-IETD fmk (20 *μ*M, 2 h), z-DEVD-fmk (20 *μ*M, 2 h) or ZB4 (a Fas-blocking antibody, 500 *μ*g/ml, 1 h). Cells were then washed with PBS and incubated in an anti-CM1 or MOPC antibody-coated (2.5 *μ*g/well) plate for 2 h. Cells were harvested and further incubated in RPMI-1640 medium. Sixteen hours later, the number of Annexin V-positive cells and mitochondrial membrane potential disruption were obtained as described in Materials and methods. The thin-line histogram represents the isotype control (MOPC). (A and D) The indicated percentage is the cell proportion in each bar. Results are representative of four independent experiments. (B and E) Proliferation assay and (C and F) apoptosis assay were performed as described previously. (C and F) The percentage value of apoptotic cells represents the mean value of three independent experimental results using flow cytometric analysis.

We wondered if FasL induction after CM1 ligation would trigger apoptosis in a Fas/FasL mechanism in the A549 lung cancer cells. To confirm that CM1-induced FasL would interact with constitutively expressed Fas on the cell surface, A549 cells were pre-incubated with ZB4, an antagonistic (blocking) anti-Fas antibody, for 1 h. Cells were washed thrice and then incubated on immobilized anti-CM1 or MOPC antibody for 2 h. ZB4 blocked the inhibition of growth effectively in A549 cells but not in HCC827 cells (Fig. 4B and E). ZB4 pretreatment also effectively blocked Annexin V-positive apoptotic cells in A549 cells but not HCC827 cells (Fig. 4C and F). Thus, the Fas-FasL interaction appeared to be active only in the A549 cells following CM1 ligation.

### ROS and caspase participates in CM1-mediated apoptosis

Both A549 and HCC827 cells were pre-incubated with z-VAD-fmk, a broad caspase inhibitor, and NAC, a scavenger of reactive oxygen species, for 2 h before ligation of CM1. Both z-VAD-fmk and NAC pretreatment blocked effectively the growth inhibition, induction of Annexin V positive apoptotic cells and mitochondrial membrane potential disruption in both lung cancer cells (Fig. 4A, B, D and E). The more selective caspase inhibitors (z-DEVD-fmk, caspase-3 inhibitor or z-IETD-fmk, a caspase-8 inhibitor) blocked CM1-mediated apoptosis in A549 cells, however, only z-DEVD-fmk blocked apoptosis of HCC827 cells (Fig. 4C and F).

### CM1-induced apoptosis activates different caspases in the two cell lines

**Figure 5.**
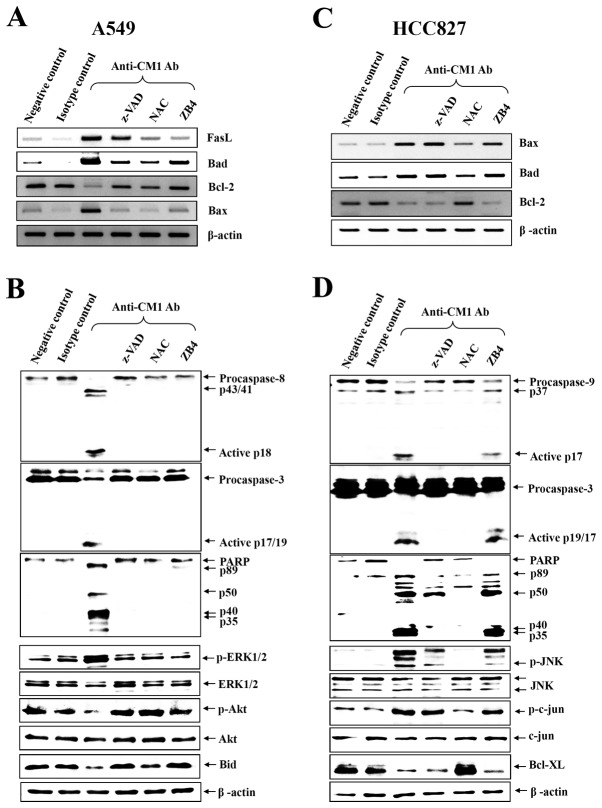
Reverse transcription polymerase chain reaction (RT-PCR) and western blot analysis for apoptotic associated genes and signaling proteins in A549 and HCC827 cells after ligation of CM1. Cells (1×10^6^ cells/ml) were pre-incubated with Z-VAD-fmk (20 *μ*M), NAC (10 mM) for 2 h or ZB4 (a Fas-blocking antibody, 500 *μ*g/ml) for 1 h. The cells were then washed with PBS and further incubated (5×10^5^ cells/well, 200 *μ*l) with anti-CM1 or isotype antibody-coated (2.5 *μ*g/well) plates for 1 h as the same condition as described in the previous experiments. Total-RNA was extracted and cDNA was synthesized for RT-PCR. (A and C) RT-PCR was performed to investigate transcription of FasL, Bax, Bad and Bcl-2. The other cells (1×10^6^ cells/ml) were pre-incubated with or without the inhibitors (Z-VAD, NAC and ZB4) for western blot analysis in the same conditions as described above. The cells were washed with PBS and further triggered (5×10^4^ cells/well, 200 *μ*l) by anti-CM1 antibody (2.5 *μ*g/well) for 1 h. The total cell lysates were obtained from these cells as described in Materials and methods. (B and D) Western blot analysis was performed for caspase, PARP activation and MAPK activation after ligation of CM1.

To elucidate the role of the different caspases in induction of apoptosis via CM1 ligation, caspase-3, -8, -9 and poly-[ADP]-ribose]-polymerase (PARP) were analyzed by western blot analysis. As shown in Fig. 5B and D, CM1 ligation induced caspase -3, -8 and PARP processing in A549 cells, however, caspase -3, -9 and PARP were processed in HCC827 cells. Furthermore, pretreatment with z-VAD-fmk, NAC and anti-Fas antagonist (ZB4) completely prevented caspase and PARP processing in A549 cells but only ZB4 blocked effectively caspase and PARP processing in HCC827 cells (Fig. 5B and D).

### CM1 ligation induces changes in pro-apoptotic and anti-apoptotic gene expression

To investigate the mechanism of CM1-mediated apoptosis by mitochondrial membrane disruption, some candidate signaling molecules were studied. Expression of genes associated with apoptosis was studied by RT-PCR in A549 and HCC827 lung cancer cells after anti-CM1 mAb or MOPC (isotype control antibody) treatment. At baseline, BCL-2 mRNA was constitutively and strongly expressed in both A549 and HCC827 cells, but BAX and BAD were slightly expressed on these cells. Following CM1 ligation, BCL-2 decreased and BAX and BAD increased. Consistent with the previous results, z-VAD-fmk, NAC and ZB4 also almost completely blocked the change on the expression of apoptosis-associated genes by CM1 ligation in A549 cells. But only NAC blocked effectively the change of the expression of apoptosis-associated genes by CM1 ligation in HCC827 cells (Fig. 5A and C).

### CM1 ligation induces the changes of MAPK phosphorylation by different mechanisms in A549 and HCC827 cells

Because apoptosis-related genes can be regulated by upstream kinases, we next examined phosphorylation of kinases after CM1 ligation in A549 and HCC827 cells. CM1 ligation induced the phosphorylation of ERK1/2, whereas it downregulated the Akt and Bid phosphorylation in A549 cells (Fig. 5B). However, CM ligation induced the phosphorylation of JNK and its major substrate c-jun, whereas it downregulated the Bcl-X_L_ expression in HCC827 cells (Fig. 5D). Furthermore, we investigated the blocking effects of various inhibitors. Z-VAD-fmk, NAC and ZB4 almost completely blocked CM1-induced phosphorylation of MAPKs in A549 cells. NAC only blocked effectively CM1-induced phosphorylation of MAPKs and downregulation of Bcl-X_L_ expression in HCC827 cells (Fig. 5B and D).

### Cytochrome c, AIF and endoG are released from mitochondria to the cytoplasm by CM1 ligation

**Figure 6.**
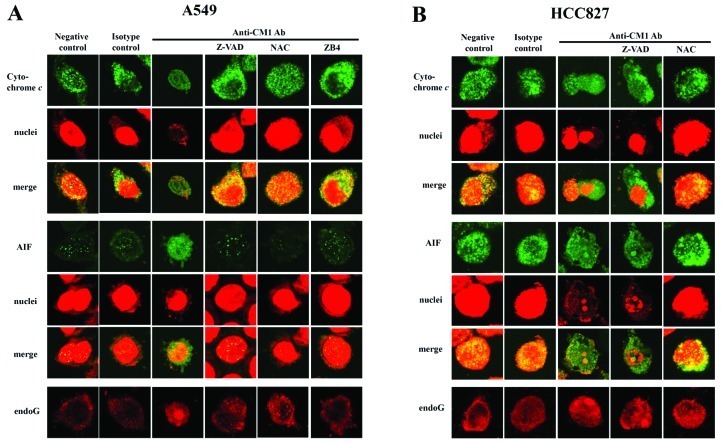
Subcellular distribution of cytochrome *c*, AIF and endoG in (A) A549 and (B) HCC827 lung cancer cells after ligation of CM1. Cells were pretreated with z-VAD-fmk (20 *μ*M, 2 h), antagonistic blocking anti-fas Ab ZB4 (0.5 *μ*g/ml, 1 h) or NAC (10 *μ*M, 1 h). Cells were washed and incubated with anti-CM1 mAb (5 *μ*g/ml, 40 min) or MOPC21 (isotype control antibody, 5 *μ*g/ml, 40 min) and goat anti-mouse IgG (2 *μ*g/ml, 15 min). Cells were harvested and further incubated in RPMI-1640 medium for 16 h. Cells were permeabilized with 0.1% saponin in PBS. Intracellular staining was performed using anti-cytochrome *c* (mouse IgG2b), AIF (mouse IgG2b) or endoG (goat polyclonal IgG) Ab and FITC-conjugated goat anti-mouse IgG or FITC conjugated rabbit anti-goat IgG. The nucleus was stained with PI. Cells were observed under a confocal microscope (×400 magnification). The procedure is described in detail in Materials and methods. Green fluorescence indicates cytochrome *c* or AIF, respectively, and red fluorescence indicates nucleus or endoG (last row).

CM1 ligation induced disruption of the mitochondrial membrane potential, so we next investigated the translocation of proapoptotic proteins in mitochondria using confocal microscopy. At baseline, in both A549 and HCC827 cells, cytochrome *c*, AIF and endoG were located within mitochondria (Fig. 6A and B, 1st and 2nd columns). After CM1 ligation in both A549 and HCC827 cells, cytochrome *c* and AIF were released from the mitochondria to the cytosol and endoG and AIF were translocated into the nucleus (Fig. 6A and B, 3rd column). In accord with previous results, in A549 cells, z-VAD-fmk, NAC and ZB4 almost completely blocked CM1-induced release of pro-apopotic proteins from the mitochondria (Fig. 6A, 4th–6th column) whereas in HCC827 cells, release was blocked only by NAC (Fig. 6B, 4th and 5th column).

## Discussion

CM1 was newly defined as a centroblast (or centrocyte) cell marker, but mainly identified as an apoptosis triggering molecule in several B lymphoma cell lines and EBV-transformed B cells (14–16). Interestingly, both flow cytometric and confocal microscopic results showed that CM1 was expressed on the cell surface in A549 and HCC827 lung cancer cells in this study. These results suggest that CM1 could be developed as a candidate marker of lung cancer for diagnosis and/or prognostic application.

The role of CM1 expressed on two lung cancer cell lines was investigated using an anti-CM1 antibody. As shown in Fig. 2, the ligation of CM1 using immobilized anti-CM1 antibody inhibited proliferation and induced the apoptosis of both A549 and HCC827 cells. CM1-mediated apoptosis involved mitochondria membrane potential disruption and intra-cellular reactive oxygen species (ROS) generation. ROS are important messengers of intracellular signaling, transcription activation, proliferation and apoptosis (17). It has long been recognized that ROS are generated by external oxidative stress or by the byproducts of altered cellular metabolism involving several oxidases such as NAD(P)H-oxidase, mitochondrial respiration or cytoskeletal organization (18,19). However, the precise mechanism of ROS generation remains unclear. ROS can modulate MAP protein kinases, cytoskeletal metabolism and intracellular Ca^2+^, and influence the mitochondrial membrane directly or indirectly (20). These studies supported that ROS had a close relationship with the mitochondrial membrane potential disruption in the mechanism of CM1-mediated apoptosis on lung cancer cells.

To evaluate the relationship with Fas-FasL signaling in CM1-mediated apoptosis, flow cytometric analysis was performed for the changes of Fas and FasL expression after the ligation of CM1. Fas (CD95) was constitutively expressed on both A549 and HCC827 cells, but FasL (CD137) was not expressed before stimulation of CM1. The ligation of CM1 using immobilized anti-CM1 antibody induced FasL expression in only A549 cells as shown in Fig. 3A. RT-PCR for FasL transcripts confirmed the fact that CM1 ligation induced FasL mRNA expression on A549 cells in Fig. 5A.

To clarify that FasL induced by CM1 ligation would interact with Fas on adjacent cells resulting in apoptosis, antagonistic anti-Fas antibody, ZB4, was used. Pretreatment of ZB4 almost completely blocked CM1-mediated apoptosis. NAC pretreatment completely blocked FasL transcription after CM1 ligation in Fig. 5A. This result indicated that FasL expression was strongly related to ROS generated by the ligation of CM1. These results suggested that ligation of CM1 induced ROS generation, and ROS triggered Fas ligand expression in A549 cells. It has already been reported that H_2_O_2_ induces upregulation of Fas and FasL expression in the nerves is linked to modulation by cAMP (21). This study also supports that CM1-mediated ROS generation leads to FasL expression.

Interestingly, ligation of CM1 did not induce Fas ligand expression in HCC827 cells despite apoptosis induction by ligation of CM1. This (Fig. 3B) result indicated that apoptosis signaling by ligation of CM1 could be triggered through different mechanisms from Fas-Fas ligand pathway in HCC827 cells. Recently, it was demonstrated that knockdown of Fas specifically enhanced cell death induced by the EGFR tyrosine kinase inhibitor in EGFR-mutant lung cancer cells (10) and Fas signaling promotes tumor growth through the JNK and Jun pathway (22). These studies also support that ligation of CM1 could induce apoptosis of HCC827 cells through another pathway rather than Fas-Fas ligand pathway.

To identify the intracellular signaling mechanism of CM1-mediated apoptosis on A549 and HCC827 lung cancer cells, further experiments using some inhibitors were performed. z-VAD-fmk, as a broad caspase inhibitor, was pre-incubated before stimulation of CM1 because caspases are commonly linked to pro-apoptotic molecules released from disrupted mitochondria, and it effectively blocked CM1-mediated apoptosis of cells and mitochondrial membrane potential disruption as expected in both A549 and HCC827 cells. Pre-treatment with NAC also completely blocked CM1-mediated apoptosis of cells and mitochondrial membrane potential disruption (Fig. 4A and D). Proliferation assay showed the same effects as the apoptosis assay results. These results suggested that both caspase activation and ROS generation was directly related to CM1-mediated apoptosis in both lung cancer cells. Additionally, z-DEVD-fmk, an executor caspase-3 inhibitor, and z-IEVD-fmk, a caspase-8 inhibitor, restored CM1-mediated apoptosis, and cleavage of procaspase-8, procaspase-3 and PARP were found after ligation of CM1 in A549 cells (Fig. 5B). However, in HCC827 cells only z-DEVD-fmk restored CM1-mediated apoptosis, and cleavage of procaspase-9, procaspase-3 and PARP were found after ligation of CM1 (Fig. 5D). Based on these results, we concluded that CM1-mediated apoptosis resulted from activation of caspases in both A549 and HCC827 lung cancer cells, but the participating caspases differed.

Bcl-2 family proteins exert many of their effects when they locate to the mitochondrial outer membrane (23). Overexpression of Bcl-2 or Bcl-X_L_ inhibits apoptosis by blocking the release of cytochrome *c*(24,25). On the contrary, Bax targeted to mitochondria can trigger rapid release of cytochrome *c*(26). Bad can heterodimerize with Bcl-X_L_ at mitochondrial membrane sites to promote cell death (27). Therefore, the expression of Bcl-2, Bax, Bad mRNA was investigated in CM1-mediated apoptosis. As expected, the expression of both Bax and Bad mRNA increased, but Bcl-2 mRNA was repressed in both lung cancer cells. Consistent with the previous results, NAC, z-VAD-fmk and ZB4 effectively blocked the changes of Bcl-2 family mRNA expression by ligation of CM1 in A549 cells, however, only NAC effectively blocked these changes in HCC827 cells (Fig. 5A and C). Thus, we concluded that the expression of the Bcl-2 family also related to CM1-mediated apoptosis in lung cancer cells.

The mitogen-activated protein kinase (MAPK) family proteins are known as the important regulators in cell apoptosis (28,29). Therefore, we evaluated whether JNK, ERK and Akt were involved in CM1-mediated apoptosis. In general, p38 MAPK and JNK are involved in cell death, whereas ERK1/2 is associated with cell proliferation. In particular, p38 MAPK is known to play a critical role in the transmission of apoptotic signals (30). In this study, ligation of CM1 induced apoptosis of A549 cells through phosphorylation of ERK1/2, whereas it induced apoptosis of HCC827 cells mainly through the phosphorylation of JNK and its major substrate c-jun. We investigated immunoblotting for p38MAPK, ERK1/2, Akt and JNK in both A549 and HCC827 cells simultaneously (data not shown partly), but we showed only positive phosphorylation results. Although the studies on correlation between Fas-Fas ligand upregulation and ERK1/2 phosphorylation are controversial, some studies show that Fas and Fas ligand proteins can be upregulated via p38 MARK/ERK activation (31,32). In our study, ligation of CM1 induced Fas ligand expression and ERK phosphorylation simultaneously in A549 cells, so we concluded that Fas ligand expression was related to ERK phosphorylation. On the other hand, ligation of CM1 induced JNK and c-jun phosphorylation independent of Fas/Fas ligand pathway in HCC827 cells. It is reported that EGFR inhibitor, AG1478, induced apoptosis via caspase-3 and JNK activation in PC-9 non-small lung cancer cells (33). Interestingly, ligation of CM1 induced apoptosis of HCC827 cells with mutated EGFR via caspase-3 and JNK activation. These results supported that CM1 could be developed as potential therapeutic target to lung cancer cells independent of EGFR mutation. Consistent with the previous results, z-VAD-fmk, NAC and ZB4 completely blocked CM1 induced the phosphorylation of MAPKs in A549, while only the NAC effectively blocked the changes of MAPKs activation by ligation of CM1 in HCC827 cells.

To determine the direct relationship between mitochondrial events and apoptosis, we investigated whether proapoptotic molecules that were released from the mitochondria and caspases were directly involved in apoptosis after ligation of CM1. We showed that proapoptotic molecules, such as cytochrome *c*, AIF and endoG, were released from the mitochondria into the cytosol after ligation of CM1 in both A549 and HCC827 cells. In addition, translocation into the nuclei of both AIF and endoG, known as executors of caspase-independent apoptosis (34), was investigated. As expected, z-VAD-fmk, NAC, ZB4 pretreatment completely blocked the release of cytochrome *c*, AIF and endoG after CM1 ligation in A549 cells, while only NAC blocked the release of these molecules in HCC827 cells. These results also support that CM1-mediated apoptosis was controlled at the mitochondrial level.

Based on these results, we conclude that ligation of CM1 induced apoptosis of A549 cells through ROS generation, FasL expression, caspase-3 and -8, mitochondrial, Bcl-2 family molecules, ERK1/2 and the Akt kinase-dependent pathways, whereas ligation of CM1 induced apoptosis of HCC827 cells was induced through ROS generation, mitochondrial, Bcl-2 family molecules, caspase-3 and -8, and the JNK and c-jun-dependent pathways. These results suggest that CM1 can be developed as one candidate for therapeutic targeting of lung cancer regardless of mutant EGFR.

## Abbreviations:

CM1: centrocyte/-blast marker 1
EGFR: epidermal growth factor receptor
